# Onset and remodeling of coronal imbalance after selective posterior thoracic fusion for Lenke 1C and 2C adolescent idiopathic scoliosis (a pilot study)

**DOI:** 10.1186/s13013-017-0123-1

**Published:** 2017-05-12

**Authors:** Masayuki Ishikawa, Kai Cao, Long Pang, Nobuyuki Fujita, Mitsuru Yagi, Naobumi Hosogane, Takashi Tsuji, Masafumi Machida, Shinichi Ishihara, Makoto Nishiyama, Yasuyuki Fukui, Masaya Nakamura, Morio Matsumoto, Kota Watanabe

**Affiliations:** 1 Spine and Spinal Cord Center, Mita Hospital, International University of Health and Welfare, 1-4-3 Mita, Minato-ku, Tokyo 108-8329 Japan; 2 Department of Orthopedic Surgery, School of Medicine, Keio University, 35 Shinanomachi, Shinjuku-ku, Tokyo 160-8582 Japan; 3 Department of Orthopedic Surgery, Murayama Medical Center, 2-37-1 Gakuen, Musashimurayama-shi, Tokyo 208-0011 Japan; 4 Department of Orthopedic Surgery, National Defense Medical College, 3-2 Namiki, Tokorozawa-shi, Saitama 359-8513 Japan; 5 Department of Orthopedic Surgery, Kitasato University, Kitasato Institute Hospital, 5-9-1 Shirokane, Minato-ku, Tokyo 108-8642 Japan; 6 Keio Spine Research Group, 35 Shinanomachi, Shinjuku-ku, Tokyo 160-8582 Japan

**Keywords:** Adolescent idiopathic scoliosis, Lenke 1C, Selective thoracic fusion, Posterior spinal fusion, Coronal balance, Coronal imbalance, Coronal decompensation, Remodeling, Surgical outcome, SRS-22

## Abstract

**Background:**

Postoperative coronal imbalance is a significant problem after selective thoracic fusion for primary thoracic and compensatory lumbar curves in adolescent idiopathic scoliosis (AIS). However, longitudinal studies on postoperative behavior of coronal balance are lacking. This multicenter retrospective study was conducted to analyze factors related to onset and remodeling of postoperative coronal imbalance after posterior thoracic fusion for Lenke 1C and 2C AIS.

**Methods:**

Twenty-one Lenke 1C or 2C AIS patients, who underwent posterior thoracic fusion ending at L3 or above, were included with a minimum 2-year follow-up. The mean patients’ age was 15.1 years at the time of surgery. Radiographic measurements were performed on Cobb angles of the main thoracic (MT) and thoracolumbar/lumbar (TLL) curves and coronal balance. Factors related to the onset of immediately postoperative coronal decompensation (IPCD) and postoperative coronal balance remodeling (PCBR), defined as an improvement of coronal balance during postoperative follow-up, were investigated using comparative and correlation analyses.

**Results:**

Mean Cobb angles for the MT and TLL curves were 57.3° and 42.3° preoperatively and were corrected to 22.8° and 22.5° at final follow-up, respectively. Mean preoperative coronal balance of −3.8 mm got worse to −21.2 mm postoperatively, and regained to −12.0 mm at final follow-up. Coronal decompensation was observed in two patients preoperatively, in ten patients immediately postoperatively, and in three patients at final follow-up. The preoperative coronal balance and lowest instrumented vertebra (LIV) selection relative to stable vertebra (SV) were significantly different between patients with IPCD and those without. PCBR had significantly negative correlation with immediately postoperative coronal balance.

**Conclusions:**

IPCD after posterior thoracic fusion for Lenke 1C and 2C AIS was frequent and associated with preoperative coronal balance and LIV selection. However, most patients with IPCD regained coronal balance through PCBR, which was significantly associated with immediately postoperative coronal balance. A fixation more distal to SV shifted the coronal balance further to the left postoperatively.

## Background

Selective thoracic fusion (STF) has been the gold standard for treating primary thoracic and compensatory lumbar curves in adolescent idiopathic scoliosis (AIS), in which both the thoracic and lumbar curves cross midline, and the lumbar curve is smaller and more flexible than the thoracic curve, since Moe had advocated its concept [1, 2]. For this curve pattern, STF induces spontaneous lumbar curve correction and preserves more mobile lumbar segments than does fusing both the thoracic and lumbar curves [3–8]. However, postoperative coronal imbalance is a significant problem after STF [7, 9–11], which may result in poor surgical outcomes with re-operation. Moreover, reported surgical outcomes have shown that patients with resultant coronal imbalance after surgery tend to have inferior Scoliosis Research Society (SRS) questionnaire score on patients’ satisfaction with treatments, compared to balanced patients [12]. Thus, the postoperative behavior of coronal balance is a major concern in the surgical outcomes for primary thoracic and compensatory lumbar curves in AIS. Causative factors reported for postoperative coronal decompensation include excessive correction of the thoracic curve, improper selection of the lowest instrumented vertebra (LIV), pre-existing coronal decompensation, and inappropriate curve identification [7–11, 13]; however, many of previous studies on postoperative behavior of coronal balance after STF are based on various surgical approaches including anterior approach and/or posterior approach with pedicle screw (PS), hook or hybrid constructs [3, 4, 6, 10, 13], and those with PS construct are scant. Apparently, surgical procedures and corrective maneuvers would influence the postoperative course of unfused lumbar curve and coronal balance.

On the other hand, we also experience some improvement of postoperative coronal imbalance during postoperative period after STF; however, some patients persist coronal imbalance. Compared to causative factors for the onset of postoperative coronal decompensation, the factors associated with the improvement of postoperative coronal imbalance during postoperative period have not been well investigated.

Thus, the purposes of this study were (1) to evaluate the postoperative behavior of coronal balance after posterior thoracic fusion with PS construct for Lenke 1C and 2C AIS (Lenke 1C/2C-AIS), in which the thoracolumbar/lumbar (TLL) curve bends less than 25° on side-bending without 20° or more kyphosis between T10 and L2, and the apical vertebra of TLL curve does not touch the center sacral vertical line (CSVL) and (2) to identify factors related to the onset of immediately postoperative coronal decompensation (IPCD) and changes of coronal balance during the follow-up period. In this study, postoperative change of coronal balance (PCCB) was defined as the change of coronal balance between preoperative and immediately postoperative evaluations, whereas postoperative coronal balance remodeling (PCBR) was defined as the improvement of postoperative coronal balance between immediately postoperative and final follow-up evaluations.

We hypothesized that preoperative radiographic measurements, LIV selection, and amount of main thoracic (MT) curve correction would have an impact on IPCD, PCCB, and PCBR.

## Methods

This study was designed as a multicenter retrospective study reviewing radiographs and clinical charts. After institutional review board approval by the ethics committee at the International University of Health and Welfare (No. 5-15-19), radiographic measurements and clinical charts review were conducted. Twenty-one patients (twenty females, one male), who underwent posterior thoracic fusion for Lenke 1C or 2C AIS at three institutes, were enrolled in this study. Inclusion criteria were as follows: (1) primary surgery by posterior thoracic fusion with PS construct of LIV ending at L3 or above for Lenke 1C/2C-AIS and (2) patients with minimum 2 years follow-up.

The mean age and Risser grade at the time of surgery were 15.1 ± 2.7 (11–22) years old and 3.4 ± 1.4 (1–5), respectively. The mean follow-up period was 3.1 (2–7.3) years. Investigated clinical data were Lenke classification, number of fused vertebrae, and level of LIV. Radiographic coronal measurements included the Cobb angles of the proximal thoracic (PT), MT, and TLL curves; apical vertebral translations (AVT) of the MT (AVT-MT) and TLL (AVT-TLL) curves; LIV tilt; coronal balance; and trunk shift on preoperative, postoperative (between 1 and 4 weeks after surgery), and final follow-up standing posteroanterior (PA) radiographs. Preoperative curve flexibilities were evaluated on right and left side-bending films. Radiographic sagittal measurements included the proximal thoracic kyphosis between T2 and T5, thoracic kyphosis between T5 and T12, thoracolumbar kyphosis between T10 and L2, lumbar lordosis between T12 and S1, and sagittal balance on preoperative, postoperative, and final follow-up standing lateral radiographs.

Measurements for coronal curvature were performed by the Cobb method. AVT-MT and AVT-TLL were measured with reference to the C7 plumb line or the CSVL, respectively. Coronal balance was measured as the horizontal distance between the C7 plumb line and the CSVL, and trunk shift was measured as the horizontal distance between the center of apex for MT curve and the CSVL. Coronal balance and trunk shift were defined as a negative value when the C7 plumb line or the center of apex for MT curve locates at the left side to the CSVL. Sagittal balance was measured as the horizontal distance between the C7 plumb line and superior-posterior corner of S1 vertebra and was defined as a positive value when the C7 plumb line locates anteriorly to superior-posterior corner of S1 vertebra, and vice versa. Coronal decompensation was defined as the absolute value of coronal balance greater than 2 cm. The ratios of values of MT curve to those of TLL curve in preoperative Cobb angle and AVT were also calculated. Reference vertebrae including the lower end vertebra (EV) and the lower neutral vertebra (NV) of MT curve, the stable vertebra (SV) at thoracolumbar lesion, and the apex of TLL curve (ApexTLL) were recorded, and the gap differences between the LIV and the reference vertebrae were counted (LIV-EV, LIV-NV, LIV-SV, ApexTLL-LIV).

Preoperative radiographic measurements, including the Cobb angles, and flexibilities of the PT, MT, and TLL curves, the AVT of the MT and TLL curves, the ratios of the MT curve to TLL curve in the Cobb angle and AVT, coronal balance, trunk shift and sagittal values, LIV selection, and postoperative correction of MT curve were evaluated whether correlating or not, to PCCB and PCBR.

Additionally, patient-reported clinical outcomes with Scoliosis Research Society-22 (SRS-22) questionnaire scores at the final follow-up were reported.

### Surgical procedure

All patients were operated on posteriorly. After exposure of the posterior elements of the spine, removal of inferior facet joints to be fused in all cases was performed. Pedicle screws were inserted bilaterally by free-hand technique, with additional attachment of some hooks in seven cases. For most patients, the LIV was selected at the SV on a PA radiograph; for others, the LIV was selected at a more distal level to partially correct the TLL curve. Corrective maneuver includes rod rotation on the concave side, followed by in-situ rod contouring, placement of a rod on the convex side, and segmental compression and distraction via PS. Intra-operative spinal cord monitoring with motor-evoked potential was routinely performed. Bone grafting was carried out using local bone materials from facetectomies and osteotomies in all cases.

### Statistical analyses

Statistical analyses were performed using the software package IBM SPSS Statistics 22 (IBM Japan, Tokyo, JAPAN). Radiographic measurement values at different time points were evaluated by analysis of variance. Unpaired *t* test was used to compare continuous variables. Correlation analyses were performed using Pearson’s correlation coefficients. A *P* value less than 0.05 was set to be statistically significant.

## Results

**Fig. 1:**
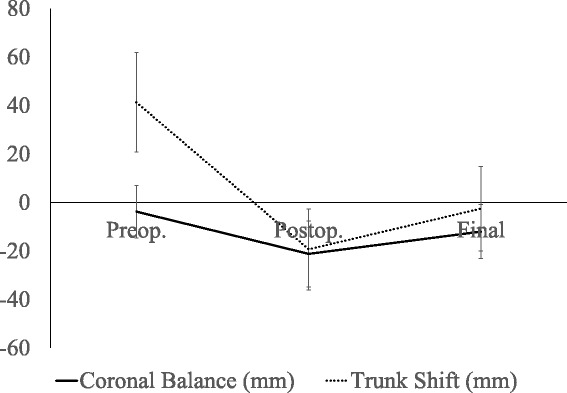
Postoperative behavior of coronal balance and trunk shift. Preoperative coronal balance and trunk shift were significantly shifted to the left immediately postoperatively; however, both of them regained balance at final follow-up

The Lenke classification was 1C− in three, 1CN in ten, 1C+ in one, 2C− in two, and 2CN in five. The mean number of fused vertebrae was 9.6 ± 2.4(6–14), and the LIV was T11 in six, T12 in six, L1 in five, L2 in three, and L3 in one (Table 1). Details of each patient are shown at Table 2. The mean Cobb angles for the PT, MT, and TLL curves were 29.4 ± 9.4° (flexibility, 24.7 ± 19.3%), 57.3 ± 12.1° (flexibility, 29.6 ± 16.3%), and 42.3 ± 5.7° (flexibility, 75.0 ± 14.6%) preoperatively and were corrected to 16.1 ± 7.6°, 17.8 ± 7.7°, and 19.3 ± 7.4° immediately postoperatively and 16.9 ± 7.8° (42.8 ± 18.8% correction), 22.8 ± 8.9° (60.4 ± 12.2% correction), and 22.5 ± 7.8° (46.6 ± 17.6% correction) at the final follow-up, respectively (Table 3). The preoperative Cobb angle ratio of the MT curve to the TLL curve was 1.4 ± 0.2. The postoperative changes in the Cobb angles of the PT, MT, and TLL curves were statistically significant (Table 3). The mean AVT-MT and AVT-TLL were 45.2 ± 15.5 mm and 22.0 ± 5.7 mm preoperatively and were corrected to 2.0 ± 11.7 mm and 25.0 ± 7.0 mm immediately postoperatively and 10.0 ± 13.0 mm and 16.8 ± 8.4 mm at the final follow-up (Table 3). The preoperative AVT ratio of the MT curve to the TLL curve was 2.4 ± 1.2. The mean coronal balance and trunk shift were −3.8 ± 10.8 mm and 41.3 ± 20.5 mm preoperatively and were corrected to −21.2 ± 13.6 mm and −19.3 ± 16.7 mm immediately postoperatively and −12.0 ± 11.1 mm and -2.5 ± 17.4 mm at the final follow-up, respectively (Table 3) (Fig. 1). Coronal decompensation was observed in two patients preoperatively, in ten immediately postoperatively, and in three at the final follow-up. No patients required revision surgery. The mean LIV tilt was 24.4 ± 12.3° preoperatively and was corrected to 8.1 ± 6.7° immediately postoperatively and 10.2 ± 5.8° at the final follow-up. The postoperative changes in AVT-MT, AVT-TLL, LIV tilt, coronal balance, and trunk shift at the final follow-up were statistically significant (Table 3).

**Table 1:** Patients’ demographics

Characteristics	*N* or mean (SD/range)
Gender	Female 20, male 1
Age (years old)	15.1 ± 2.7
Risser grade	3.4 ± 1.4
Follow-up period (years)	3.1 (2–7.3)
Lenke classification	1C− (3)
	1CN (10)
	1C+ (1)
	2C− (2)
	2CN (5)
Mean fused vertebrae	9.6 ± 2.4
Lowest instrumented vertebra	T11 (6)
	T12 (6)
	L1 (5)
	L2 (3)
	L3 (1)

**Table 2:** Details of each patient

Case no.	Age (y/o)	Gender	Follow-up period (years)	Lenke Classification	Fusion level	MT curve (°)				TLL curve (°)				Coronal balance (mm)		
						Level	Preop.	Postop.	Final	Level	Preop.	Postop.	Final	Preop.	Postop.	Final
1	12	F	4.0	1CN	T4–T12	T6–T11	52	10	13	T11–L3	47	33	19	−9	−22	−16
2	11	F	5.5	2CN	T3–L1	T5–T11	76	32	39	T11–L4	45	17	20	0	−5	−10
3	15	F	7.3	1CN	T2–L2	T6–T12	60	22	24	T12–L4	42	22	25	10	−5	0
4	14	F	5.0	1C–	T2–L2	T6–T11	55	20	18	T11–L3	32	14	15	−10	−53	−26
5	14	F	4.5	1CN	T2–L1	T5–T11	52	15	12	T11–L4	38	20	26	−12	−22	−2
6	14	F	3.0	1CN	T2–L3	T5–T11	51	15	18	T11–L4	44	25	37	−2	−37	−18
7	17	F	2.0	1CN	T3–L2	T5–T10	47	14	14	T10–L4	44	13	13	−2	−41	−40
8	13	F	5.0	2CN	T5–T12	T6–T12	51	28	28	T12–L4	43	31	32	−22	−30	−33
9	20	F	2.0	2CN	T2–L1	T5–T11	92	33	36	T11–L4	51	13	26	−10	−27	−2
10	12	F	2.3	2CN	T2–T12	T5–T12	76	18	38	T12–L4	55	18	29	14	−8	0
11	14	F	2.3	2C−	T4–T11	T5–T11	50	8	13	T11–L4	42	9	6	−13	−6	−17
12	13	F	2.6	2C−	T5–T11	T5–T11	49	7	17	T11–L3	38	8	17	−25	−26	−17
13	17	M	2.3	2CN	T5–L1	T5–T12	52	14	25	T12–L4	32	25	17	11	−12	−10
14	18	F	2.0	1CN	T5–T12	T5–T11	61	22	21	T11–L4	41	26	26	0	−18	−11
15	15	F	2.0	1CN	T6–T11	T5–T12	39	14	24	T12–L4	40	14	27	−4	−24	−9
16	17	F	2.0	1CN	T5–T12	T5–T11	48	13	16	T11–L4	34	24	22	−14	−44	−11
17	22	F	2.8	1C−	T5–L1	T4–T12	65	20	21	T12–L4	43	13	12	0	−14	−6
18	17	F	3.1	1C−	T3–T11	T3–T11	54	30	32	T11–L4	44	32	31	0	−15	−16
19	15	F	2.3	1CN	T5–T12	T5–T12	58	9	13	T12–L4	48	15	16	−8	−15	−12
20	13	F	2.1	1C+	T5–T11	T4–T12	64	18	35	T12–L4	46	14	32	0	−8	4
21	15	F	2.0	1CN	T5–T11	T4–T11	50	13	23	T11–L4	40	19	26	15	−13	0

*MT* main thoracic, *TLL* thoracolumbar/lumbar

**Table 3:** Radiographic coronal measurements

Coronal measurements	Preop.	Postop.	Final follow-up
PT curve (°)	29.4 ± 9.4	16.1 ± 7.6^a^	16.9 ± 7.8^a^
Flexibility (%)	24.7 ± 19.3		
Correction rate (%)			42.8 ± 18.8
MT curve (°)	57.3 ± 12.1	17.8 ± 7.7^a^	22.8 ± 8.9^a^
Flexibility (%)	29.6 ± 16.3		
Correction rate (%)			60.4 ± 12.2
TLL curve (°)	42.3 ± 5.7	19.3 ± 7.4^a^	22.5 ± 7.8^a^
Flexibility (%)	75.0 ± 14.6		
Correction rate (%)			46.6 ± 17.6
AVT-MT (mm)	45.2 ± 15.5	2.0 ± 11.7^a^	10.0 ± 13.0^a^
AVT-TLL (mm)	22.0 ± 5.7	25.0 ± 7.0	16.8 ± 8.4^a^
LIV tilt (°)	24.4 ± 12.3	8.1 ± 6.7^a^	10.2 ± 5.8^a^
Coronal balance (mm)	−3.8 ± 10.8	−21.2 ± 13.6^a^	−12.0 ± 11.1^a^
Trunk shift (mm)	41.3 ± 20.5	−19.3 ± 16.7^a^	−2.5 ± 17.4^a^

Values indicate mean ± standard deviation

*PT* proximal thoracic, *MT* main thoracic, *TLL* thoracolumbar/lumbar, *AVT* apical vertebral translation, *LIV* lowest instrumented vertebra

^a^Statistical significance

Preoperative sagittal measurements did not change significantly at the final follow-up (Table 4).

**Table 4:** Radiographic sagittal measurements

Sagittal measurements	Preop.	Postop.	Final follow-up
T2–T5 (°)	8.8 ± 4.7	11.6 ± 5.1	11.3 ± 5.9
T5–T12 (°)	17.5 ± 10.8	15.8 ± 8.1	18.2 ± 8.1
T10–L2 (°)	−4.9 ± 12.4	−2.8 ± 10.1	−4.0 ± 9.5
T12–S1 (°)	−53.1 ± 12.0	−44.6 ± 12.5	−48.1 ± 10.6
Sagittal balance (mm)	−9.7 ± 23.3	3.2 ± 22.0^a^	−17.1 ± 13.5

Values indicate mean ± standard deviation

^a^Statistical significance

The mean gap differences in LIV-EV, LIV-NV, LIV-SV, and ApexTLL-LIV were 1.0 ± 1.4, 1.1 ± 1.5, 0.6 ± 1.4, and 1.9 ± 1.2, respectively.

### Factors associated with IPCD, PCCB, and PCBR

In 20 of 21 patients, coronal balance shifted to the left immediately after surgery. Comparative study showed that among preoperative radiographic measurements, LIV selection and the amount of MT curve correction, the preoperative coronal balance and trunk shift and LIV-SV were significantly different between patients with IPCD and those without (−11.0 ± 7.8 mm vs 2.7 ± 9.0 mm, *p* < 0.01; 30.1 ± 15.1 mm vs 51.5 ± 19.9 mm, *p* < 0.05; 1.3 ± 1.5 vs −0.1 ± 0.9, *p* < 0.05, respectively) (Table 5). PCCB had significant correlation to the LIV selection (LIV-EV *r* = −0.58, *p* < 0.01; LIV-NV *r* = −0.47, *p* < 0.05; LIV-SV *r* = −0.48, *p* < 0.05; ApexTLL-LIV *r* = 0.61, *p* < 0.01), whereas PCCB had no significant correlation to the preoperative radiographic measurements or the amount of MT curve correction (Table 6).

**Table 5:** Comparative analyses between patients with IPCD and those without

Parameters		IPCD (*N* = 10)	Non-IPCD (*N* = 11)	*P* value
PT Cobb (°)	Preop.	27.8 ± 7.2	30.7 ± 11.2	0.50
	Flexibility (%)	15.7 ± 12.8	31.7 ± 21.3	0.10
	Postop.	16.8 ± 8.3	15.5 ± 7.4	0.72
	Final	16.5 ± 6.3	17.3 ± 9.2	0.81
	Correction rate (%)	41.3 ± 17.3	44.3 ± 20.8	0.72
MT Cobb (°)	Preop.	53.6 ± 14.2	60.5 ± 9.2	0.20
	Flexibility (%)	29.2 ± 18.9	29.9 ± 14.7	0.93
	Postop.	16.9 ± 7.9	18.7 ± 7.7	0.59
	Final	19.6 ± 7.8	25.8 ± 9.1	0.11
	Correction rate (%)	63.2 ± 12.4	57.9 ± 12.1	0.33
TLL Cobb (°)	Preop.	41.1 ± 5.8	43.5 ± 5.6	0.36
	Flexibility (%)	76.2 ± 16.2	74.2 ± 14.4	0.81
	Postop.	19.5 ± 8.5	19.1 ± 6.7	0.90
	Final	23.3 ± 7.6	21.7 ± 8.2	0.65
	Correction rate (%)	43.0 ± 17.2	49.8 ± 18.2	0.39
AVT-MT (mm)	Preop.	41.1 ± 16.2	48.8 ± 14.7	0.27
	Postop.	3.9 ± 13.3	0.0 ± 10.2	0.47
	Final	9.3 ± 12.1	10.6 ± 14.3	0.84
AVT-TLL (mm)	Preop.	24.1 ± 6.7	20.1 ± 4.2	0.13
	Postop.	27.2 ± 7.7	23.1 ± 6.1	0.21
	Final	18.5 ± 9.7	15.3 ± 7.4	0.43
LIV tilt (°)	Preop.	18.6 ± 11.2	29.2 ± 11.4	0.05
	Postop.	7.8 ± 7.2	8.4 ± 6.5	0.87
	Final	9.8 ± 6.1	10.5 ± 5.7	0.78
Coronal balance (mm)	Preop.	**−11.0 ± 7.8**	**2.7 ± 9.0**	**<0.01**
	Postop.	**−32.5 ± 10.7**	**−10.8 ± 4.6**	**<0.01**
	Final	**−17.4 ± 12.5**	**−7.1 ± 7.1**	**<0.05**
Trunk shift (mm)	Preop.	**30.1 ± 15.1**	**51.5 ± 19.9**	**<0.05**
	Postop.	**−28.6 ± 17.4**	**−10.8 ± 11.0**	**<0.05**
	Final	**−**9.0 ± 15.9	3.5 ± 17.2	0.10
LIV-EV		1.6 ± 1.7	0.5 ± 0.9	0.09
LIV-NV		1.5 ± 1.8	0.7 ± 1.1	0.24
LIV-SV		**1.3 ± 1.5**	**−** **0.1 ± 0.9**	**<0.05**
ApexTLL-LIV		1.4 ± 1.3	2.3 ± 1.0	0.10

Values indicate mean ± standard deviation. Bold values indicate a statistical significance

*IPCD* immediately postoperative coronal decompensation, *PT* proximal thoracic, *MT* main thoracic, *TLL* thoracolumbar/lumbar, *AVT* apical vertebral translation, *LIV* lowest instrumented vertebra, *EV* end vertebra, *NV* neutral vertebra, *SV* stable vertebra

**Table 6:** Correlation analyses on PCCB and PCBR

Parameters		PCCB		PCBR	
		*r*	*P* value	*r*	*P* value
PT Cobb	Preop.	0.27	0.23	**−**0.20	0.39
	Flexibility	0.02	0.93	**−**0.13	0.63
	Postop.	**−**0.04	0.87	**−**0.05	0.84
	Final	0.16	0.48	**−**0.14	0.54
	Correction rate	0.16	0.50	**−**0.09	0.71
MT Cobb	Preop.	0.16	0.49	0.05	0.84
	Flexibility	0.13	0.59	0.02	0.92
	Postop.	**−**0.01	0.95	**−**0.04	0.86
	Final	0.10	0.66	**−**0.11	0.65
	Correction rate	0.00	0.99	0.15	0.53
TLL Cobb	Preop.	0.27	0.24	**−**0.28	0.22
	Flexibility	0.02	0.95	0.02	0.96
	Postop.	**−**0.14	0.54	**−**0.09	0.72
	Final	**−**0.16	0.48	0.28	0.23
	Correction rate	0.28	0.22	**−**0.41	0.07
AVT-MT	Preop.	0.12	0.62	0.16	0.49
	Postop.	**−**0.03	0.90	**−**0.12	0.62
	Final	0.16	0.50	**−**0.24	0.30
AVT-TLL	Preop.	**−**0.14	0.57	0.31	0.20
	Postop.	0.25	0.30	0.07	0.77
	Final	0.16	0.50	**−**0.02	0.93
LIV tilt	Preop.	0.18	0.44	**−**0.27	0.25
	Postop.	**−**0.46	0.05	0.03	0.89
	Final	**−**0.38	0.10	0.12	0.62
Coronal balance	Preop.	**−**0.33	0.14	**−**0.12	0.60
	Postop.	**0.66**	**<0.01**	**−0.61**	**<0.01**
	Final	0.26	0.26	0.25	0.28
Trunk shift	Preop.	**−**0.09	0.71	0.06	0.81
	Postop.	**0.51**	**<0.05**	**−0.57**	**<0.01**
	Final	0.32	0.15	0.01	0.98
LIV-EV		**−0.58**	**<0.01**	0.24	0.29
LIV-NV		**−0.47**	**<0.05**	0.18	0.44
LIV-SV		**−0.48**	**<0.05**	0.17	0.47
ApexTLL-LIV		**0.61**	**<0.01**	**−**0.31	0.17

A *r* indicates Pearson’s correlation coefficient. Bold values indicate a statistical significance

*PCCB* postoperative change of coronal balance, *PCBR* postoperative coronal balance remodeling, *PT* proximal thoracic, *MT* main thoracic, *TLL* thoracolumbar/lumbar, *AVT* apical vertebral translation, *LIV* lowest instrumented vertebra, *EV* end vertebra, *NV* neutral vertebra, *SV* stable vertebra

**Fig. 2:**
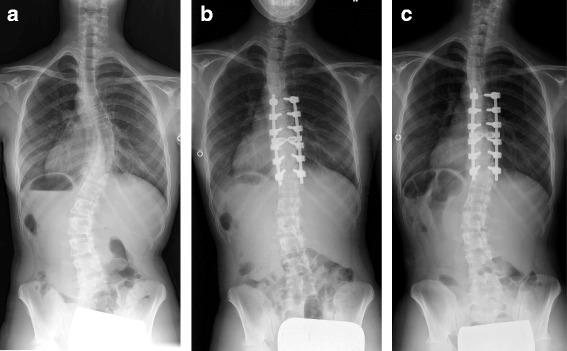
A representative case (Case No. 15). A 15-year-old female with Lenke 1CN adolescent idiopathic scoliosis underwent selective posterior thoracic fusion from T6 to T11. The preoperative coronal balance of −4 mm (**a**) worsened to −24 mm immediately after surgery (**b**), but recovered to −9 mm with remodeling, as seen 2 years after surgery (**c**)

On the contrary, PCBR had no significant correlation to the preoperative radiographic measurements, LIV selection, or the amount of MT curve correction, whereas PCBR had significant correlation to the immediately postoperative coronal balance (*r* = −0.61, *p* < 0.01) and trunk shift (*r* = −0.57, *p* < 0.01) (Table 6) (Fig. 2).

### SRS-22 questionnaire scores

Domain scores of SRS-22 questionnaire were 4.4 ± 0.2 in Pain, 4.4 ± 0.5 in Function, 3.4 ± 0.9 in Self-Image, 3.9 ± 1.0 in Mental Health, 3.9 ± 0.7 in Satisfaction, and 4.0 ± 0.4 in Total at the final follow-up.

## Discussion

The surgical outcomes for a primary thoracic curve with a compensatory lumbar curve remain one of the most controversial issues in surgical AIS treatment. Advantages of STF for this curve pattern are saving more mobile lumbar segments and inducing spontaneous lumbar curve correction; however, the risks of postoperative coronal decompensation and resultant marked lumbar curve magnitude compared to correction and fusion of both the thoracic and lumbar curves are considered to be shortcomings in STF. The primary thoracic and compensatory lumbar curves are generally classified as King type II or Lenke 1C/2C curves, in which the MT curve is larger and more rigid than the TLL curve and both the MT and TLL curves cross ﻿midline. In these curve patterns, coronal balance are usually prone to shift to the left preoperatively, and previous studies have shown that coronal balance tends to shift further to the left after STF, which may result in coronal decompensation in some patients [7]. Whereas, it is also known that patients with immediately postoperative coronal imbalance usually regain coronal balance to some degrees during the follow-up period; however, some patients persist coronal imbalance after surgery.

Several causative factors have been reported for postoperative coronal decompensation; however, many of these causative factors were based on not only various surgical approaches but also the evaluation with the change of coronal balance between preoperative and final follow-up evaluations. Detailed analyses to seek the factors associated with the changes of coronal balance between preoperative and immediately postoperative follow-ups and between immediately postoperative and final follow-ups are still lacking. PCBR, defined as the improvement of coronal balance between immediately postoperative and final follow-ups, is another aspect of the postoperative behavior of coronal balance, compared to the onset of postoperative coronal decompensation.

Thus, we attempted to define the correlative factors to IPCD, PCCB, and PCBR after posterior thoracic fusion with PS construct in Lenke 1C/2C-AIS.

As demonstrated in the results, coronal balance tends to shift to the left preoperatively, and it shifts further to the left immediately postoperatively in 20 of 21 patients. To detect the causative factors for the onset of IPCD, we performed comparative and correlation analyses. From the comparative study, preoperative coronal balance and trunk shift, and LIV-SV were found to be significantly different between compensated and decompensated patients immediately postoperatively, whereas correlation analysis found that PCCB was significantly correlated to the LIV selection relative to the EV, NV, SV, and ApexTLL. Regarding LIV selection, only the LIV-SV was found to be significantly associated with both the onset of IPCD and PCCB. These results indicate that the immediately postoperative change of coronal balance depends only on LIV selection, and not on preoperative radiographic measurements or the amount of MT curve correction, and that a fixation more distal to the SV shifts the coronal balance even further to the left immediately postoperatively. Moreover, a patient with a pre-existing coronal imbalance to the left tends to be decompensated to the left immediately postoperatively if the LIV is selected at a level more distal to the SV. It is important to note that coronal balance shifted further to the left immediately postoperatively in majority of patients even when the LIV was selected at the SV, indicating that STF may itself produce an immediately postoperative leftward shift of the coronal balance [11]. Accordingly, patients with preoperative coronal imbalance further to the left and inappropriate selection of LIV, such as fixation distal to the SV would be at a high risk of IPCD. These findings suggest that selecting the LIV at the SV would minimize the risk of IPCD for patients with Lenke 1C/2C-AIS.

Regarding PCBR, this study demonstrated that PCBR occurred in majority of patients more than 5 mm during postoperative periods and the deterioration of immediately postoperative coronal balance occurred in only limited cases (four cases) at the final follow-up and also found that PCBR had significantly negative correlation to the immediately postoperative coronal balance and trunk shift. These results indicate that patients with coronal balance shifted further to the left immediately postoperatively tend to compensate more balance during the follow-up period, which may be attributed to a postural reflex, potentially existing in the relatively flexible lumbar curves (Lenke 1C/2C-AIS), although three patients persisted coronal decompensation at the final follow-up. That is, PCBR is dependent on the immediately postoperative condition in coronal balance and trunk shift. Coronal decompensation at the final follow-up was observed in three patients, so comparative analysis to detect the causative factors for coronal decompensation at the final follow-up was not performed because of the limited number of patients, although the LIV-SV tended to be larger in patients with coronal decompensation at the final follow-up, compared to those without (2.3 vs 0.3). Patients with coronal decompensation at the final follow-up were all decompensated immediately postoperatively, and none of the compensated patients immediately postoperatively were decompensated at the final follow-up. Accordingly, optimal curve identification and surgical strategy to minimize the risk of IPCD may be more beneficial to prevent coronal decompensation at the final follow-up. The current study suggests that selecting the LIV at the SV minimizes the risk of postoperative coronal decompensation after posterior thoracic fusion using PS construct for Lenke 1C/2C-AIS and results in acceptable patients-reported outcomes.

Further studies to determine the optimal candidate for STF based on the preoperative characteristics of the TLL curve and coronal balance are needed to prevent postoperative coronal decompensation in the primary thoracic and compensatory lumbar curves.

This retrospective study has several limitations, including the small sample size and relatively short follow-up period. As for the follow-up period, observation with longer postoperative period is critical in the evaluation of clinical and radiographic outcomes, especially in treating young patients. However, the postoperative spontaneous unfused lumbar curve correction and remodeling of trunk shift after STF are reported to occur usually within 2 years after surgery [14], so a minimum 2-year follow-up observation may minimize these concerns.

## Conclusions

The onset of IPCD was associated with the preoperative coronal balance and trunk shift, and the LIV selection relative to the SV, whereas PCBR was associated with the immediately postoperative coronal balance and trunk shift in patients treated by posterior thoracic fusion using PS construct for Lenke 1C/2C-AIS. Selecting the LIV at the SV would be optimal in treating Lenke 1C/2C-AIS in terms of avoiding postoperative coronal decompensation.

## Abbreviations

AIS: Adolescent idiopathic scoliosis
AVT: Apical vertebral translation
CSVL: Center sacral vertical line
EV: End vertebra
IPCD: Immediately postoperative coronal decompensation
LIV: Lowest instrumented vertebra
MT: Main thoracic
NV: Neutral vertebra
PA: Posteroanterior
PCBR: Postoperative coronal balance remodeling
PCCB: Postoperative change of coronal balance
PS: Pedicle screw
PT: Proximal thoracic
SRS: Scoliosis Research Society
STF: Selective thoracic fusion
SV: Stable vertebra
TLL: Thoracolumbar/lumbar
